# Supplementary material for: Mariana-type ophiolites constrain the establishment of modern plate tectonic regime during Gondwana assembly
Source: Nat Commun. 2021 Jul 7;12:4189. doi: 10.1038/s41467-021-24422-z (PMC8263587; doi:10.1038/s41467-021-24422-z)
Supplement: Supplementary file 1 — Supplementary Information [file 41467_2021_24422_MOESM1_ESM.pdf]

Supporting Information for

Mariana type ophiolites constrain establishment of modern plate tectonic regime during Gondwana assembly

Jinlong Yao [1 *], Peter A. Cawood [2], Guochun Zhao [3, 1 *], Yigui Han [1], Xiaoping Xia [4], Qian Liu [3], Peng Wang [3]

[1] State Key Laboratory of Continental Dynamics, Department of Geology, Northwest University, Northern Taibai Street 229, Xi'an 710069, China

[2] School of Earth, Atmosphere & Environment, Monash University, Melbourne, VIC 3800, Australia

[3] Department of Earth Sciences, The University of Hong Kong, Pokfulam Road, Hong Kong SAR

[4] State Key Laboratory of Isotope Geochemistry, Guangzhou Institute of Geochemistry, Chinese Academy of Sciences, Guangzhou, 510640, China

corresponding authors: yaojinlong@nwu.edu.cn; gzhao@hku.hk

Contents of this file

I. Supplementary notes

1. Geology background
2. Sample description
3. Analytical methods
4. Initial subduction of the Proto-Tethys Ocean

II. Supplementary references

III. Supplementary Figures 1–8

IV. Supplementary Tables 1–5

I. Supplementary notes

1. Geology background

The northern boundary of the Altyn terrane is the northern Altyn fault zone, but its southern margin is less well constrained and is offset by the Cenozoic reactivation of the Altyn fault zone associated with India-Asia collision (1, 2). The boundary between central and south Altyn terranes is inferred to be an unnamed strike slip fault. The North Altyn Terrane contains an assemblage of Archean to Paleoproterozoic igneous and metamorphic rocks that are considered to constitute the basement to the Tarim Block, and are unconformably overlain by Mesoproterozoic strata (3, 4). The central Altyn Terrane, also referred to as the Milanhe-Jinyanshan terrane, is dominated by marble and meta-clastic sedimentary rocks that are covered by a thick layer of limestone-dolomite, assigned to the Bashikuergan and Taxidaban groups, respectively. They were inferred to be Mesoproterozoic, but latest investigations suggest Neoproterozoic to early Paleozoic ages (author unpublished data, 5, 6). The units are unconformably overlain by limestone and clastic sedimentary rocks of the early Paleozoic Suoerkuli Group. Voluminous early Paleozoic granitoids dated at 522-430 Ma, mostly S-type, intrude into these sequences (6-9). Meta-mafic arc sequences dated at ca. 520-510 Ma occur within the northern margin of the terrane (author unpublished data). The Southern Altyn Terrane consists primarily of a suite of granitic gneisses and meta-sedimentary rocks referred to as the Altyn Complex, including ortho- and paragneisses, marbles, amphibolites, and minor meta- mafic-ultramafic rocks (4). This complex was earlier considered to be Archean-Paleoproterozoic in age (4), but recent works indicate the complex contains latest Mesoproterozoic to early Paleozoic units (5, 8, author unpublished data), which experienced multiple stages of metamorphism and deformation at 500-430 Ma, as well as events at, or after, ca. 235 Ma (1-2, 10-12). An inferred ophiolitic mélange (the Munabulake ophiolite) of early Paleozoic age is also present in the complex. HP-UHP metamorphic rocks occur as lenses within the Altyn Complex, including UHP eclogite and kyanite-garnet bearing pelitic gneiss, HP-UHP

garnet lherzolite (10-12). These HP/UHP rocks yield metamorphic ages of ca. 509-475 Ma, with their protolith age constrained to ca. 840-750 Ma (10). Granitic plutons with variable compositions (I-, S- and A- types) intrude into the Altyn Complex. Limited dating on these intrusions yields ages of 462-440 Ma (e.g. 6-9). The southern Altyn ophiolite belt and the Hongliugou-Lapeiquan ophiolite belt are inferred to be fragments of the Proto-Tethys Ocean (11, 13). The Hongliugou-Lapeiquan ophiolitic belt consists mainly of sheared and deformed ophiolitic rock units of both supra-subduction zone (SSZ) and mid-ocean ridge basalt (MORB) affinities, and are dated at 518 ± 4 Ma, 513 ± 3 Ma, and 479 ± 8 Ma (SHRIMP zircon U-Pb and LA-ICP-MS zircon U-Pb; 14-15). Ophiolite components include serpentinized harzburgite and lherzolite, mafic-ultramafic cumulates, sheeted dykes, and pillow basalt (14). The South Altyn ophiolite belt, extending more than 700 km along strike from Mangya to Tula, lies within the Altyn fault zone and contains ophiolite mélange and flysch. The sheared ophiolite sequences are not well preserved and occur as disrupted blocks of serpentinized dunite, harzburgite and some gabbroic rock, dated at 510 ± 1 and 501 ± 2 Ma (9 and references therein). Adakites dated at ca. 517 Ma and 503 Ma also occur within this ophiolite belt (e.g., 9 and references therein).

2. Sample description

In this study, we selected nine metamorphosed basaltic samples 17SAT2-2, 17SAT5, 17SAT7-1, 17SAT7-6, 17SAT8, 18SAT32-1, 18SAT33-2, 18SAT36-2 and 18SAT36-3, and eight samples of more intermediate composition (17SAT2-1, 17SAT3-1, 17SAT3-2, 17SAT9, 17SAT10, 17SAT11-2-1, 17SAT11-2-2, and 17SAT11-4) from the Munabulake ophiolite for analysis. Collected ultramafic rock samples include harzburgite (17SAT22-1, 17SAT14, and 18SAT41-4) and an olivine pyroxenite (18SAT41-5) (Fig. s1f, s1g, s1h). Meta-gabbro sample 17SAT13-2 was collected for age dating. Although earlier mapping indicates dunites are present in the ophiolite, they are not accessible as they occur within a newly established restricted access nature reserve.

The serpentinized harzburgite samples 17SAT13-1, 17SAT14, 17SAT22-1 and 18SAT41-4, contain about 50-65 % olivine and 20-30 % orthopyroxene, along with 15 % magnetite, tremolite, and other accessory minerals (Fig. s1f), whereas the olivine pyroxenite sample 18SAT41-5 contains 35 % olivine, 40 % orthopyroxene, and 10 % clinopyroxene, and 15 % tremolite and other accessory minerals (Fig. s1g). Inclusions of olivine within orthopyroxene are also present in sample 18SAT41-4 (Fig. s1h). The foliated meta-basaltic samples are mainly composed of 25-35 % plagioclase and 35-45 % hornblende that may have been derived from metamorphism of pyroxene minerals, but primary clinopyroxene and orthopyroxene are preserved and along with magnetite constitute the remaining 15-20 % of the basalt mineralogy. Pyroxene cleavage can be observed in hornblende in the amphibolite, indicating a gabbroic protolith. Minor secondary quartz grains have also been observed. In sample 18SAT13-2, some olivine grains are present. The foliated intermediate samples mostly contain some 50 % plagioclase, 25-30 % hornblende, 8-15 % quartz, 10 % opaque oxide mineral, and accessory minerals. In addition, fine grained volcanic rocks locally occur (sample 17SAT2-1). The siliceous rocks consist predominantly of quartz and minor calcite.

3. Analytical methods

Zircon cathodoluminescence (CL) images were obtained to reveal the internal texture of the grains, preparing for further zircon U-Pb age analyses and Hf-O isotopes, as well as water contents. It was conducted on Mono CL3+ (Gatan, U.S.A.) attached to a Quanta 400 FEG electron microscope at the State Key Laboratory of Continental Dynamics in Northwest University. Zircon U-Pb age analysis was carried out using Agilent Technologies 7700x quadrupole inductively coupled plasma mass spectrometry system (ICP-MS; Hachioji, Tokyo, Japan) equipped with a 193 nm ArF excimer laser ablation system (LA; Bozeman, Montana, USA). Laser with 8 Hz frequency and energy of 6.0 J/cm2 were applied, with an ablation spot of 33 μm. Zircon standard 91500 was used to calibrate U-Pb isotopic ratios (16), and two zircon standards GJ-1 and Plešovice were analyzed as unknown, after every eight or nine sample spots (17-18). Raw data

were processed by using software ICPMSDataCal (19). Detailed analytical results are presented in Table s1.

zircon Lu-Hf isotopic analysis was carried out using Nu Plasma II multi-collector ICP-MS (MC-ICP-MS; Wrexham, Wales, UK) attached to a 193 nm ArF excimer LA ablation system (Bozeman, Montana, USA). During lab analysis, 8 Hz frequency and 40 μm spot diameter was applied for all the zircon samples. Zircon standards Plešovice, GJ-1, 91500, Mud Tank, and Penglai were analyzed after analysis every ten sample spots to ensure instrument stability and for data calibration (20). Detailed analytical results are presented in Table s2.

Zircon oxygen isotopes and water contents were simultaneously analyzed on the CAMECA IMS 1280-HR at the GIGCAS (Guangzhou Institution of Geochemistry, Chinese Academy of Sciences), SIMS lab. Detailed procedures are comparable to those described by Xia et al. (2019) (21). The analytical area for all the zircons is 30 × 30 μm. A Cs+ primary beam of 3 to 4 nA with energy of 10 Kv was applied to sputter secondary ions from analyzed zircon samples. The isotope data were normalized to the reference material 91500 ($\delta^{18}O$ VSMOW = 9.9 ± 0.3 ‰, 1SD; VSMOW, Vienna standard for mean ocean water; 22). The external precision estimated by repeating measurement of the 91500 Standard was 0.45 ‰ (2SD, n = 45). Detailed analytical results are presented in Table s3.

Analysis of bulk rock trace element composition was carried out at Nanjing FocuMS Technology Co. Ltd. 40mg powder of each sample was dissolved with1.0ml HF and 0.5ml HNO_3, in high-pressure PTFE containers. The containers were then steel-jacketed and placed in constant temperature oven with a given temperature at 195°C. Mafic samples were placed in the oven for 48h for and 72h for felsic samples. Diluent of samples was then analyzed using an Agilent Technologies *7700x* quadrupole ICP-MS machine (Hachioji, Tokyo, Japan) to determine their trace element compositions, which gives precision better than ±10% for elements >10ppm and better than 5% for the elements >50ppm. The measured elements were calibrated using USGS standards for basalt (BIR-1, BCR-2, BHVO-2). Detailed analytical results are presented in Table s4.

The elemental compositions analysis for the olivine and spinel were carried out using a JEOL 8230 EMPA at Xi'an Center of CGS (China Geology Survey). Accelerating voltage and specimen current were maintained at 15 kV and 10 nA during analysis, with beam diameter of 5 μm and counting times of 10 s on-peak and 5 second on-background for all elements. The following mineral standards were used, including Cr2O3 (Cr Kα), hematite (Fe Kα), apatite (P Kα, Ca Kα), olivine (Mg Kα, Si Kα), jadeite (Al Kα, Na Kα), rutile (Ti Kα), NiO (Ni Kα), orthoclase (K Kα), and rhodonite (Mn Kα). Detailed analytical results are presented in Table s5.

4. Initial subduction of the Proto-Tethys Ocean

Early Paleozoic ophiolites and trench-arc assemblages in continental blocks of East Asia are related to the evolution of the Proto-Tethys Ocean and accretion of these blocks to the northern Gondwana margin (e.g. 13, 23-24). The Proto-Tethys Ocean has been given a number of localized names adjacent to the variety of continental and arc related blocks inferred to lie within the ocean (e.g. 24-29). The overall subduction initiation of this ocean is not constrained and inferred individual subduction zones have largely been treated in isolation.

The main branch of the Proto-Tethys Ocean is referred to as the east-west trending Shangdan – South Qilian – Altyn ocean, with relicts preserved across central China. The Shangdan Ocean subducted northward beneath the southern margin of the North Qinling terrane, which commenced by 514 Ma as based on the earliest gabbroic arc magmatic rocks (28 and references therein). Further west, northward subduction of the South Qilian-Altyn Ocean beneath the Qilian and Altyn terranes (24, 27) commenced by 525-520 Ma based on ages for ophiolite and intra-oceanic arc complexes (6, 8, 30-32). In addition, some 515 Ma arc volcanic rocks and 450-420 Ma UHP rocks also occur in the northern Qaidam margin (11), may be related to possible southward subduction of the South Qilian Ocean. Thus, the main branch of the Proto-Tethys Ocean, i.e. the Shangdan- South Qilian- Altyn ocean, principally subducted northward and

initiated almost simultaneously at ca. 525-515 Ma, and is analogous to widespread subduction initiation ophiolites of the West Pacific plate and Neo-Tethys Ocean (33-34).

The northern branch of the Proto-Tethys Ocean consists of a series of inferred back-arc marginal basins and includes the east-west trending Erlangping- North Qilian - North Altyn oceans, which principally subducted southward but may have experienced double divergent directed subduction. The Erlangping Ocean between the North Qinling Block and North China Craton was generated due to back-arc rifting and subducted southward at around 510 Ma (28). The relics of the north Qilian Ocean are comprised of ca. 560–450 Ma MORB and back-arc basin ophiolite suites in the northern Qilian margin (27, 31 and references therein). Andean-type continental arc (520–445 Ma) and boninitic sequence (517–490 Ma) in the northern Qilian margin (27, 35 and references therein) are related to south directed subduction initiation of the North Qilian Ocean. Further west, the north Altyn Ocean, located between the Altyn Terrane and Tarim Block, is preserved in the ca. 521-480 Ma Hongliugou-Lapeiquan ophiolite (8, 14, 36). To the south of southwestern Tarim, the 525-495 Ma Kudi back-arc ophiolite indicates southward subduction of the unnamed ocean beneath the west Kunlun terrane (25, 37). The earliest reported arc magmatism in the terrane dated at ca. 533 Ma (38). Therefore, southward subduction initiation of the northern branch of the Proto-Tethys Ocean commenced occurred at ca. 510 Ma in the east, at ca. 520 Ma in the central segment and ca. 530 Ma in the west.

The southern branch of the Proto-Tethys Ocean lies south of the South China-Indochina Block and the Qaidam Block, and to the north of the Kongtum Terrane and Qiangtang Block which were likely already accreted to the Gondwana margin (24, 26, 39). In the east segment, an unnamed ocean subducted both northward beneath the possibly linked South China-Indochina Block and southward beneath the Kongtum micro-block, with northward subduction initiation before ca. 490 Ma (40-41). Ophiolites occur in the Indochina Block, but with no well constrained age (42). In the Kunlun Ocean (Qimantag Ocean), south of Qaidam (24, 43), northward subduction is inferred to have initiated at some time before ca. 550 Ma based on ages of

metamorphism at 549-500 Ma and SSZ type ophiolite at ca. 530-460 Ma (13, 43). Further south, the ocean subducted southward beneath the Qiangtang Block and initiated at some time before ca. 535 Ma (26, 39, 44). Thus, subduction initiation of the southern branch of the Proto-Tethys Ocean possibly varies from around 550 Ma to 490 Ma.

II. Supplementary references

1. Yin, A. et al. Tectonic history of the Altyn Tagh fault system in northern Tibet inferred from Cenozoic sedimentation. *Geological Society of American. Bulletin* 114, 1257–1295 (2002).
2. Cowgill, E., Yin, A., Harrison, T.M. & Wang, X.F. Reconstruction of the Altyn Tagh fault based on U-Pb geochronology: Role of back thrusts, mantle sutures, and heterogeneous crustal strength in forming the Tibetan Plateau. *Journal of Geophysical Research: Solid Earth* 108(B7) (2003).
3. Ge, R.F. et al. Generation of Eoarchean continental crust from altered mafic rocks derived from a chondritic mantle: The ~3.72 Ga Aktash gneisses, Tarim Craton (NW China). *Earth and Planetary Science Letters* 538, 116225 (2020).
4. Bureau of Geology and Mineral Resources of Xinjiang Uygur Autonomous Region (BGMRXUAR). Regional Geology of Xingjiang Uyger Autonomous Region. Geological Publishing House, China, pp. 315–318 (in Chinese with English abstract) (1993).
5. Wang, C. et al. Provenance and ages of the Altyn Complex in Altyn Tagh: implications for the early Neoproterozoic evolution of northwestern China. *Precambrian Research* 230, 193–208 (2013).
6. Liu, L., Kang, L., Cao, Y.T. & Yang, W.Q. Early Paleozoic granitic magmatism related to the processes from subduction to collision in South Altyn, NW China. *Science China: Earth Sciences* 58, 1513–1522 (2015).
7. Liu, C. H. et al. Age, composition, and tectonic significance of Palaeozoic granites in the Altyn orogenic belt, China. *International Geology Review* 58(2), 131-154 (2016).
8. Sobel, E. R. & Arnaud, N. A possible middle Paleozoic suture in the Altyn Tagh, NW China. *Tectonics* 18(1), 64-74 (1999).
9. Kang, L. et al. Early Paleozoic magmatism and collision orogenic process of the South Altyn. *Acta Geologica Sinica* 90(10), 2527-2550 (2016).
10. Liu, L. et al. Evidence of former stishovite in UHP eclogite from the South Altyn Tagh, western China. *Earth*

and Planetary Science Letters 484, 353-362 (2018).

11. Zhang, J. X. et al. Subduction, accretion and closure of Proto-Tethyan Ocean: Early Paleozoic accretion/collision orogeny in the Altun-Qilian-North Qaidam orogenic system. *Acta Petrologica Sinica*, 31(12), 3531-3554 (2015). (in Chinese with English abstract).
12. Zhang, J.X., Yu, S.Y. & Mattinson, C.G. Early Paleozoic polyphase metamorphism in northern Tibet, China. *Gondwana Research* 41, 267-289 (2017).
13. Li, S. Z. et al. Closure of the Proto-Tethys Ocean and Early Paleozoic amalgamation of microcontinental blocks in East Asia. *Earth-Science Reviews* 186, 37-75 (2018).
14. Yang, J. S. et al. Petrology and SHRIMP age of the Hongliugou ophiolite at Milan, north Altun, at the northern margin of the Tibetan plateau. *Acta Petrologica Sinica* 24(7), 1567-1584 (2008) (in Chinese with English abstract).
15. Gao, X., Xiao, P., Guo, L., Dong, Z. & Xi, R. Opening of an early Paleozoic limited oceanic basin in the northern Altyn area: Constraints from plagiogranites in the Hongliugou-Lapeiquan ophiolitic mélange. *Science China–Earth Sciences* 54(12), 1871–1879 (2011).
16. Wiedenbeck, M. et al. Three natural zircon standards for U-Th-Pb, Lu-Hf, trace element and REE analyses. *Geostandards Newsletter* 19, 1–23 (1995).
17. Jackson, S.E. et al. The application of laser ablation–inductively coupled plasma–mass spectrometry to in situ U-Pb zircon geochronology. *Chemical Geology* 211, 47–69 (2004).
18. Sláma, J. et al. Plešovice zircon—A new natural reference material for U-Pb and Hf isotopic microanalysis. Chemical Geology 249, 1–35 (2008).
19. Liu, Y.S. et al. Reappraisal and refinement of zircon U-Pb isotope and trace element analyses by LA-ICP-MS: Chinese Science Bulletin 55, 1535–1546 (2010).
20. Morel, M.L.A. et al. Hafnium isotope characterization of the GJ-1 zircon reference material by solution and laser-ablation MC-ICPMS. *Chemical Geology* 255, 231–235 (2008).
21. Xia, X.P. et al. Zircon water content: reference material development and simultaneous measurement of oxygen isotopes by SIMS. *Journal of Analytical Atomic Spectrometry* 34, 1088-1097 (2019).
22. Wiedenbeck, M. et al. Further Characterisation of the 91500 Zircon Crystal. *Geostandards and Geoanalytical Research* 28, 9-39 (2004).
23. Huang, B., Piper, J. D., Sun, L. & Zhao, Q. New paleomagnetic results for Ordovician and Silurian rocks of the Tarim Block, Northwest China and their paleogeographic implications. *Tectonophysics* 755, 91-108 (2019).

24. Zhao, G. C. et al. Geological reconstructions of the East Asian blocks: From the breakup of Rodinia to the assembly of Pangea. *Earth-Science Reviews* 186, 262-286 (2018).

25. Xiao, W., Windley, B. F., Hao, J. & Li, J. Arc-ophiolite obduction in the Western Kunlun Range (China): implications for the Palaeozoic evolution of central Asia. *Journal of the Geological Society* 159(5), 517-528 (2002).

26. Cawood, P. A., Johnson, M. R. & Nemchin, A. A. Early Palaeozoic orogenesis along the Indian margin of Gondwana: Tectonic response to Gondwana assembly. *Earth and Planetary Science Letters* 255(1-2), 70-84 (2007).

27. Song, S. G. et al. Continental orogenesis from ocean subduction, continent collision/subduction, to orogen collapse, and orogen recycling: The example of the North Qaidam UHPM belt, NW China. *Earth-Science Reviews* 129, 59-84 (2014).

28. Dong, Y.P. & Santosh, M. Tectonic architecture and multiple orogeny of the Qinling Orogenic Belt, Central China. *Gondwana Research* 29(1), 1-40 (2016).

29. Liu, L. et al. Early Paleozoic tectonic evolution of the North Qinling Orogenic Belt in Central China: Insights on continental deep subduction and multiphase exhumation. *Earth-Science Reviews* 159, 58-81 (2016).

30. Li, X. M. et al. Characteristics and age study about the Yuemakeqi mafic-ultramafic rock in the southern Altyn Fault. *Acta Petrologica Sinica* 25(4), 862-872 (in Chinese with English abstract) (2009).

31. Song, S. et al. Qi-Qin Accretionary Belt in Central China Orogen: accretion by trench jam of oceanic plateau and formation of intra-oceanic arc in the Early Paleozoic Qin-Qi-Kun Ocean. Science Bulletin 62(15), 1035-1038 (2017).

32. Yan, Z. et al. Early Cambrian Muli arc-ophiolite complex: a relic of the Proto-Tethys oceanic lithosphere in the Qilian Orogen, NW China. *International Journal of Earth Sciences* 108(4), 1147-1164 (2019).

33. Stern, R. J. & Gerya, T. Subduction initiation in nature and models: A review. *Tectonophysics* 746, 173-198 (2018).

34. Whattam, S.A. & Stern, R.J. The ‘subduction-initiation rule’: A key for linking ophiolites, intra-oceanic forearcs and subduction initiation. *Contributions to Mineralogy and Petrology* 162, 1031–1045 (2011).

35. Xia, L. Q., Li, X. M., Yu, J. Y. & Wang, G. Q. Mid-late neoproterozoic to early paleozoic volcanism and tectonic evolution of the Qilianshan, NW China. *GeoResJ* 9, 1-41 (2016).

36. Gao, X., Xiao, P., Guo, L., Dong, Z. & Xi, R. Opening of an early Paleozoic limited oceanic basin in the northern Altyn area: Constraints from plagiogranites in the Hongliugou-Lapeiquan ophiolitic mélange. *Science China–*

Earth Sciences 54(12), 1871–1879 (2011).

37. Li, T.F. & Zhang, J.X. Zircon LA-ICP-MS U-Pb ages of websterite and basalt in Kudi ophiolite and the implication, West Kunlun. *Acta Petrologica Sinica* 30(8), 2393-2401 (2014).

38. Yin, J. et al. (2020). Petrogenesis of Early Cambrian granitoids in the western Kunlun orogenic belt, Northwest Tibet: Insight into early stage subduction of the Proto-Tethys Ocean. *Bulletin*, 132(9-10), 2221-2240.

39. Zhu, D.C. et al. The origin and pre-Cenozoic evolution of the Tibetan Plateau. *Gondwana Research 23* (4), 1429–1454 (2013).

40. Wang, Y.J. et al. 2020. Early Paleozoic subduction in the Indochina interior: Revealed by Ordo-Silurian mafic-intermediate igneous rocks in South Laos. *Lithos* 362, 105488.

41. Gardner, C.J. et al. Evidence for Ordovician subduction-related magmatism in the Truong Son terrane, SE Laos: implications for Gondwana evolution and porphyry Cu exploration potential in SE Asia. *Gondwana Research 44*, 139-156 (2017).

42. Tran, H.T. et al. The Tam Ky-Phuoc Son shear zone in Central Vietnam: tectonic and metallogenic implications. *Gondwana Research* 26(1), 144-164 (2014).

43. Dong, Y. P. et al. Subduction and accretionary tectonics of the East Kunlun orogen, western segment of the Central China Orogenic System. *Earth-Science Reviews* 186, 231-261 (2018).

44. Yin, A. & Harrison, T.M. Geologic evolution of the Himalayan–Tibetan orogen. *Annual Review of Earth and Planetary Sciences* 28, 211–280 (2000).

45. Sun, S. S. & McDonough, W. F. Chemical and isotopic systematics of oceanic basalts: implications for mantle composition and processes. *Geological Society, London, Special Publications 42(1)*, 313-345 (1989).

46. McDonough, W. F. & Sun, S. S. The composition of the Earth. *Chemical geology* 120(3-4), 223-253 (1995).

47. Miyashiro, A. Volcanic rock series in island arcs and active continental mar-gins. *American Journal of Science* 274, 321–355 (1974).

48. Pearce, J.A. Geochemical fingerprinting of oceanic basalts with applications to ophiolite classification and the search for Archean oceanic crust. *Litho*s 100,14–48 (2008)

49. Shervais, J.W. Ti–V plots and the petrogenesis of modern and ophiolitic lavas. *Earth and Planetary Science Letters* 59, 101–118 (1982).

50. Reagan, M.K. et al. Fore-arc basalts and subduction initiation in the Izu–Bonin–Mariana system. *Geochem. Geophys. Geosyst.* 11, 1–17 (2010).

51. Sorbadere, F., Schiano, P., Metrich, N. & Bertagnini, A. Small-scale coexistence of island-arc- and enriched-

MORB-type basalts in the central Vanuatu arc. *Contributions to Mineralogy and Petrology* 166(5), 1305–1321 (2013).

52. Woodhead, J.D., Eggins, S.M. & Johnson, R.W. Magma genesis in the New Britain Island Arc: Further insights into melting and mass transfer processes. *Journal of Petrology* 39, 1641–1668 (1998).

53. Stern, R. J. et al. To understand subduction initiation, study forearc crust: To understand forearc crust, study ophiolites. *Lithosphere* 4(6), 469-483 (2012).

III. Supplementary Figures 1-8

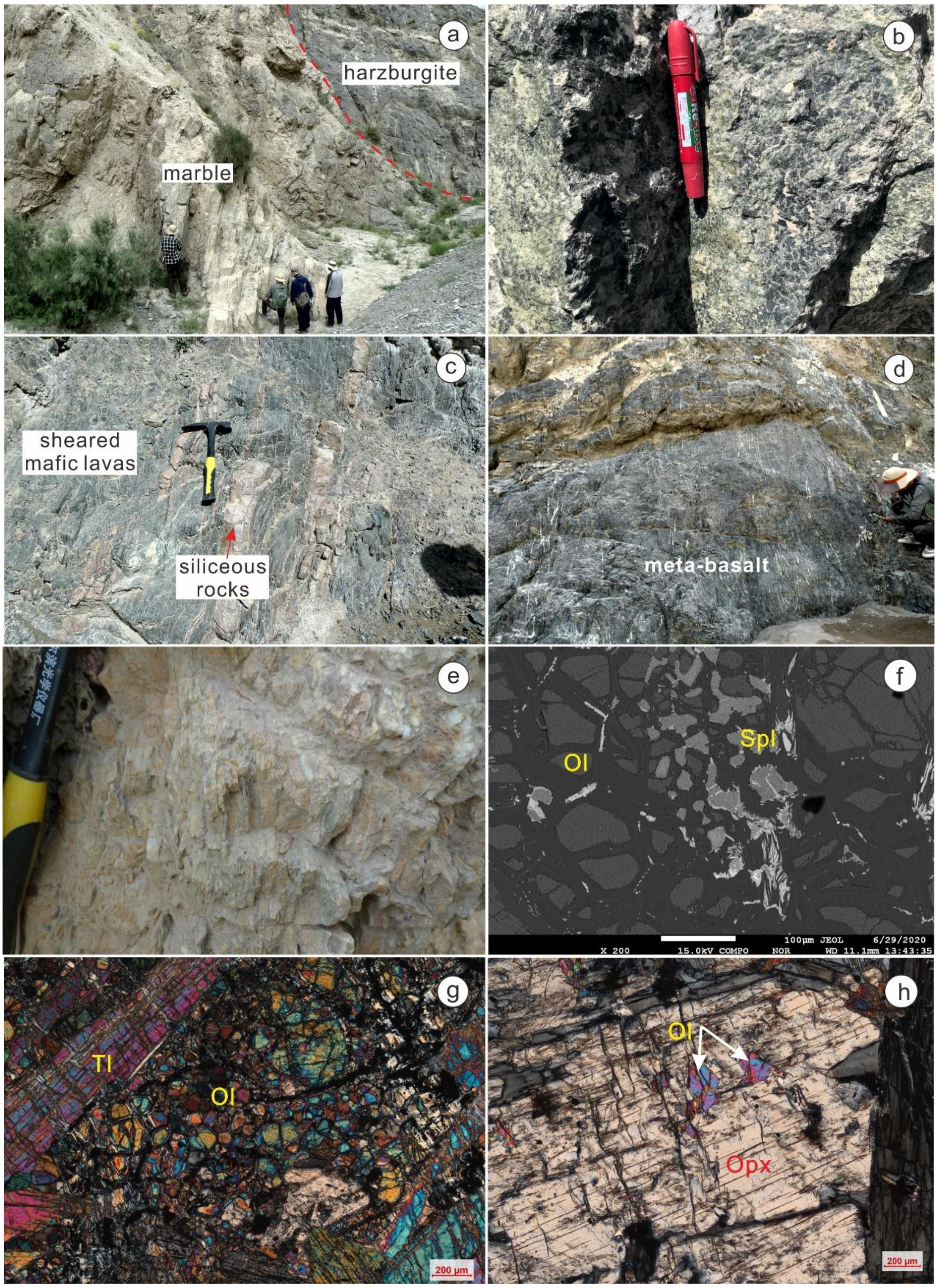

Fig. S1. Representative field photos of the Munabulake ophiolite, and thin-section photomicrographs and BSE (Electron back scattered diffraction) image of samples, (a) marble blocks within harzburgite; (b) harzburgite; (c) blocks of siliceous rocks within

meta-mafic lavas; (d) meta-mafic lavas; (e) siliceous rocks of varied thickness interlayered with sheared mafic lavas; (f) spinel in harzburgite; (g) Tremolite and olivine within olivine pyroxenite; (h) olivine with serpentinized rims observed in harzburgite. Abbreviations: Ol, olivine; Opx, orthopyroxene; Sp, spinel; Tl, tremolite.

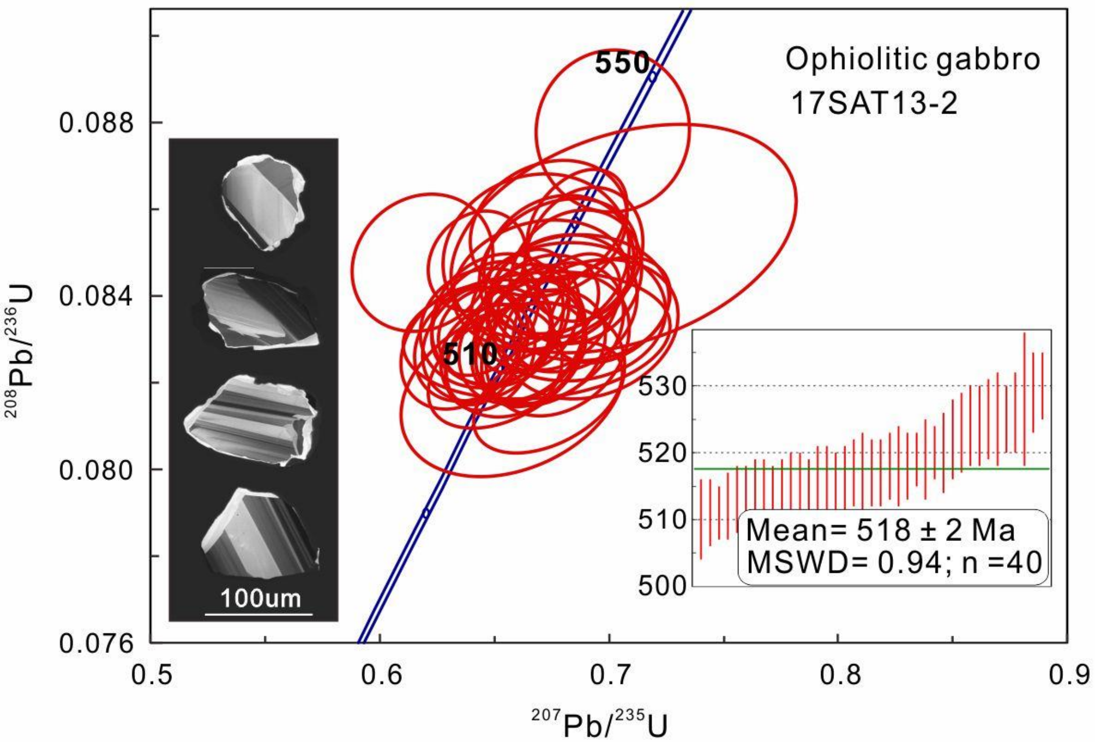

Figure S2. U-Pb concordia plots for zircons from ophiolitic gabbro 17SAT13-2. Data-point error symbols are 1s and data-point error ellipses are 68.3% confidence.

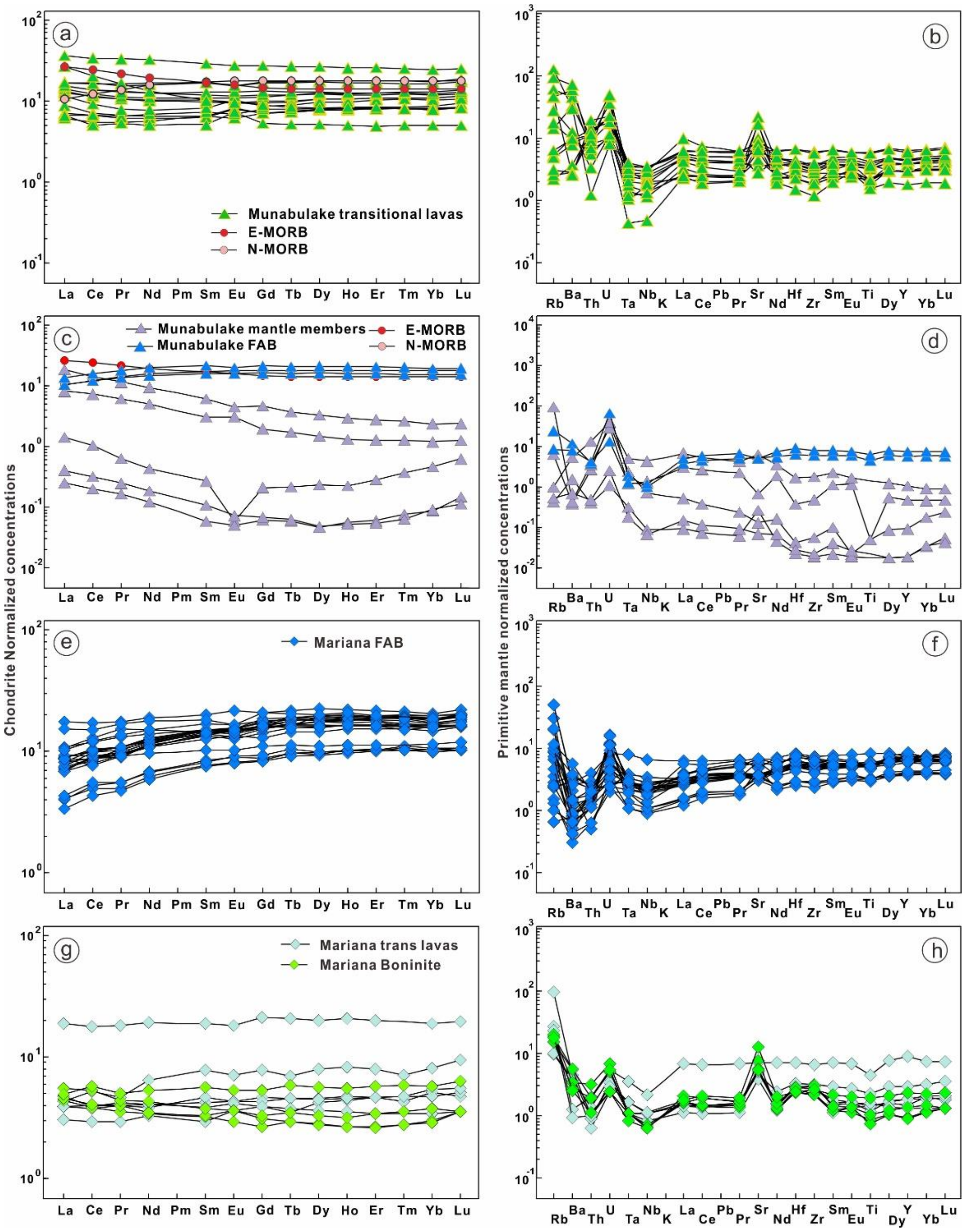

Fig. S3. Chondrite normalized REE patterns for (a) transitional lavas of the Munabulake ophiolite; (c) fore-arc basalt and residue mantle member samples of the Munabulake ophiolite; (e) fore-arc basalt of Mariana subduction initiation ophiolite; (g) boninites and transitional lavas of the Mariana subduction initiation ophiolite. Primitive mantle– normalized incompatible element distribution spidergrams for (b) transitional lavas of the Munabulake ophiolite; (d) fore-arc basalt and residue mantle member samples of the Munabulake ophiolite; (f) fore-arc basalt of the Mariana subduction initiation ophiolite, (h) boninites and transitional lavas of Mariana subduction initiation ophiolite. (The normalization values are from 45-46).

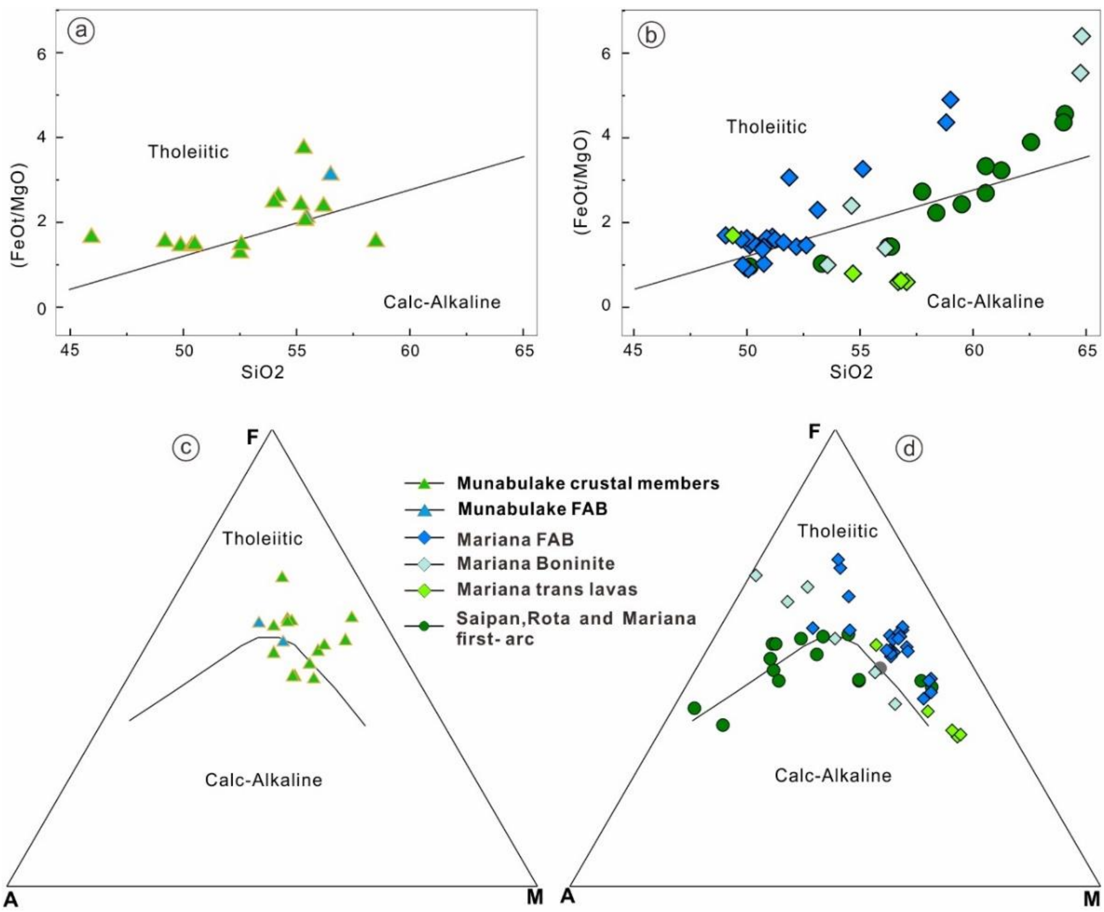

Fig. S4 Discrimination of crustal member, FAB, transitional lavas and boninites of the Munabulake ophiolite (left) and those of Mariana forearc (right) on the SiO2 vs FeOt/MgO and AFM discrimination diagrams (after 47)

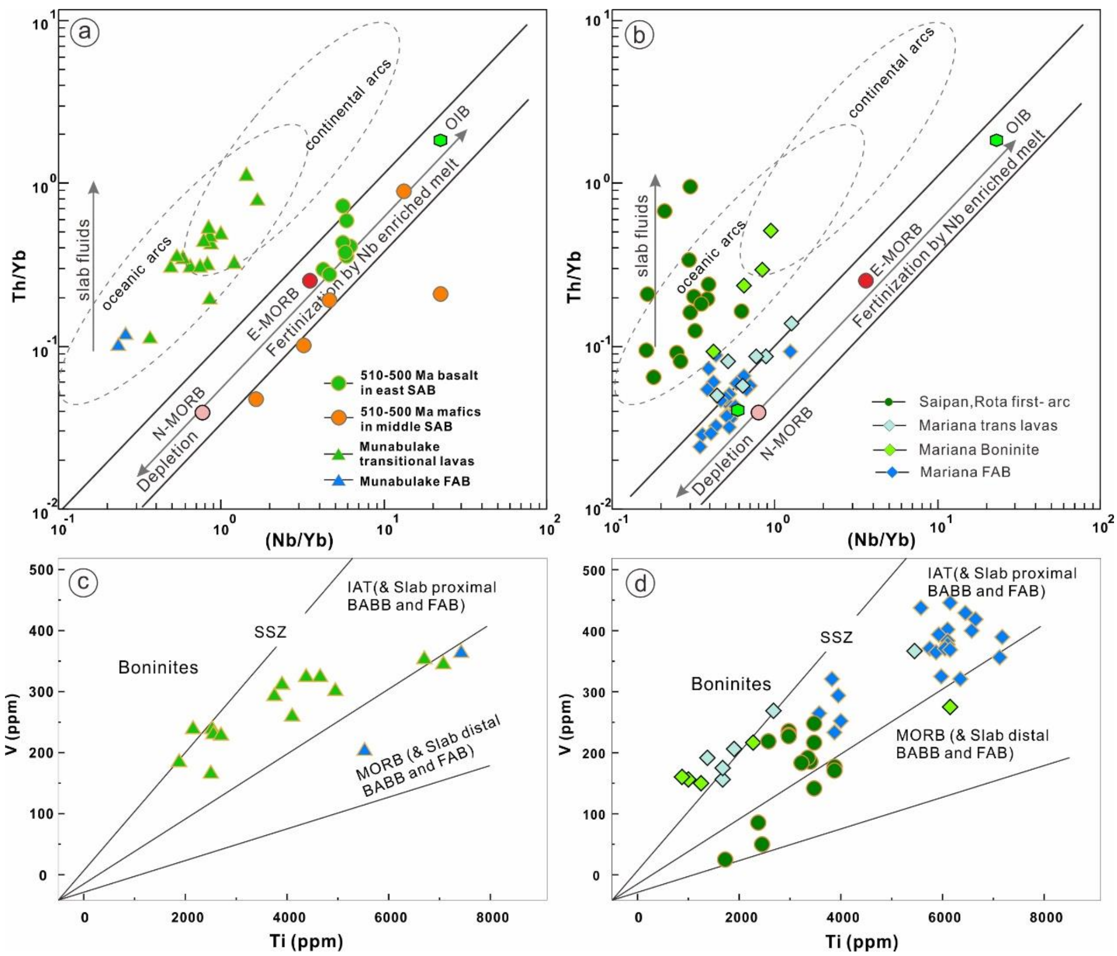

Fig. S5. Discrimination of igneous suites of the Munabulake ophiolite and South Altyn ophiolite belt (left) and those of Mariana forearc lavas (right): (a) and (b) Th/Yb-Ta/Yb diagrams (e, f, after 48); (c) and (d) Ti vs. V diagrams (a, b, after 49). Data sources: 50 for Mariana FAB, transitional lavas and boninites.

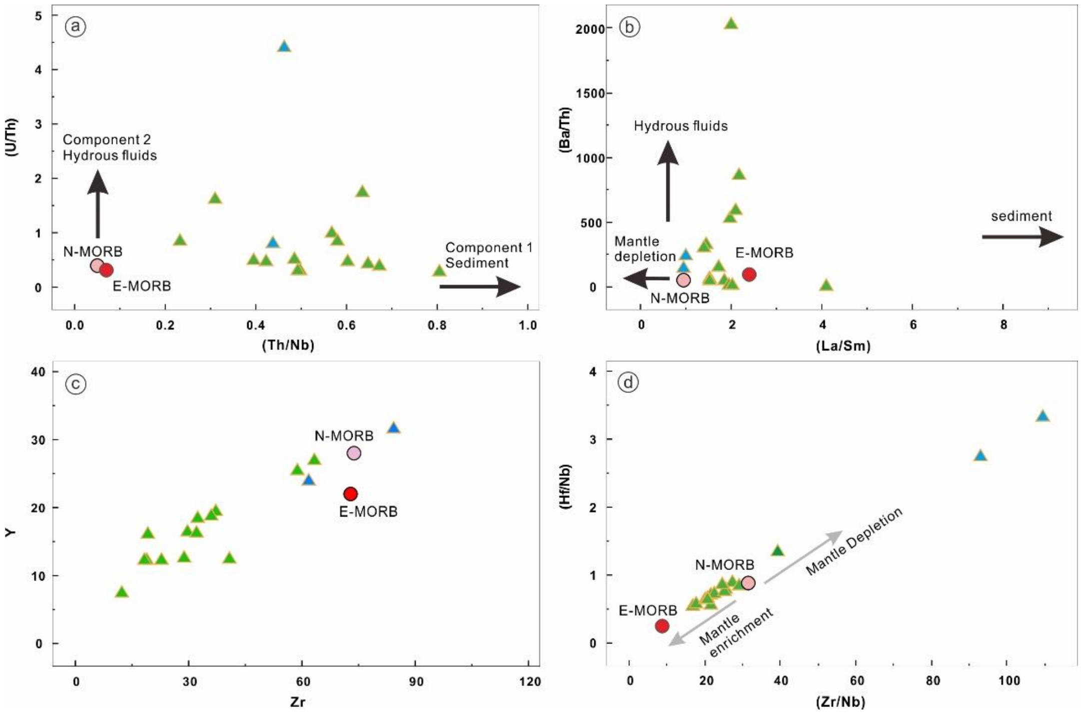

Fig. S6. (a) U/Th vs. Th/Nb; (b) Ba/Th vs La/Sm; (c) Zr vs Y; (d) Hf/Nb vs Zr/Nb diagram showing the relative contribution of subducted components (after 51-52)

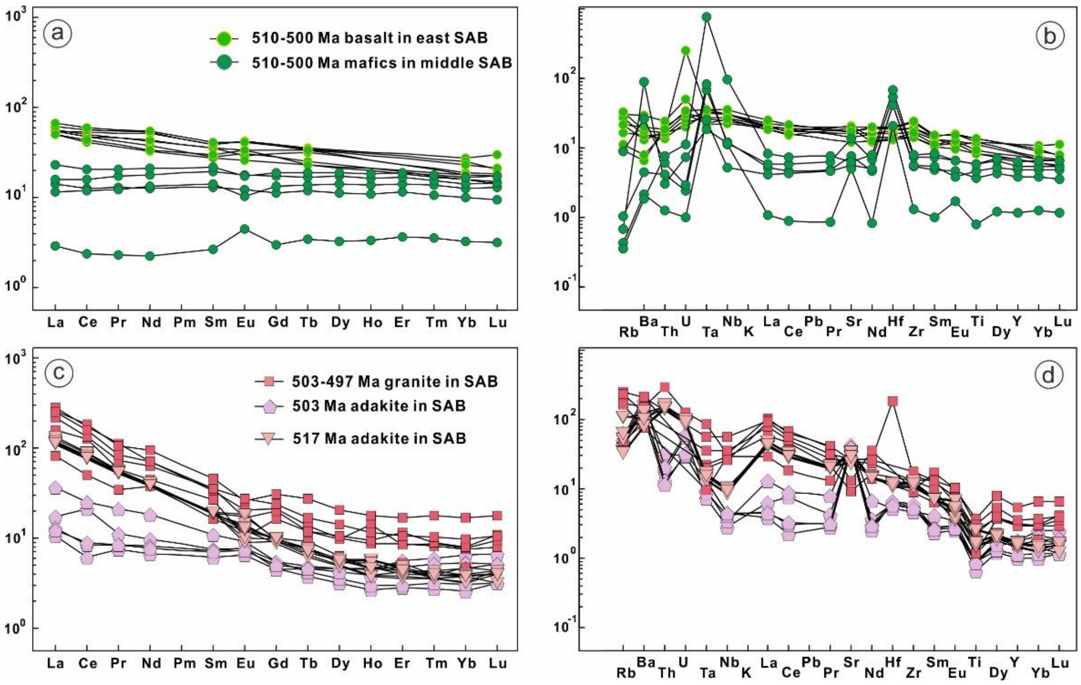

Fig. S7. (e) REE patterns and (f) incompatible element distribution spidergrams for MORB type mafic-ultramafic suites in the South Altyn ophiolite belt; (g) REE patterns and (f) incompatible element distribution spidergrams for adakites and calc-alkaline granitoids within the South Altyn ophiolite belt. (The normalization values are from 45-46).

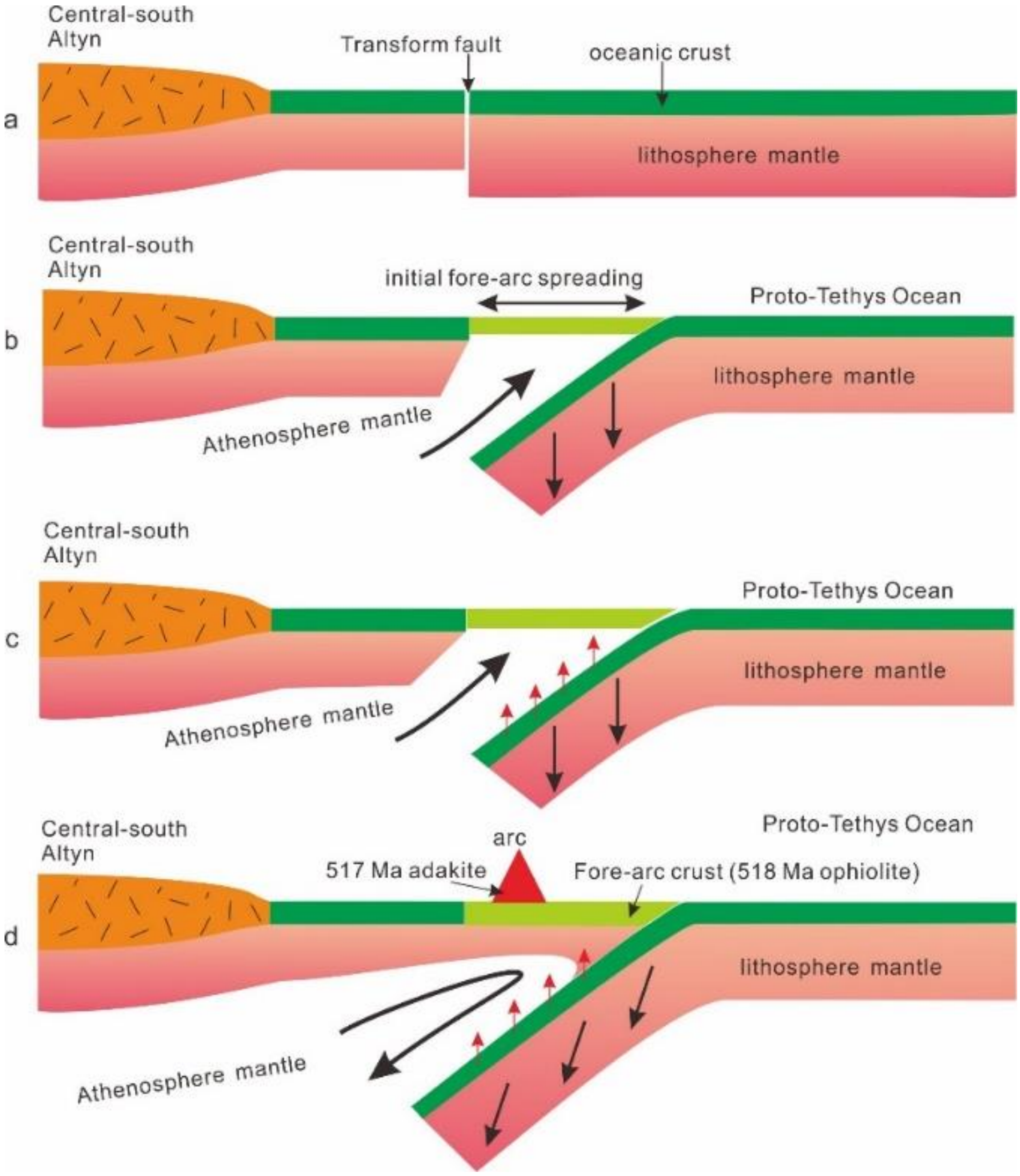

Fig. S8. Tectonic model for formation of the Munabulake subduction initiation ophiolite, based on subduction initiation model by Stern et al. (2012) (53). (a) Oceanic crust boundary (transform fault) prior to subduction initiation; (b) Upwelling of fertile asthenosphere; (c) Depleted mantle stagnates; strong interaction with slab-derived fluid; (d) Downdip motion of lithosphere signals start of true subduction, which terminates rapid trench rollback and proto-forearc spreading

Supplementary Tables 1–5

Table S1. Zircon U-Pb ages of the olivine gabbro within the Munabulake ophiolite.

	MEASURED RATIOS						CORRECTED AGES (Ma)									
Analysis	*$^{207}Pb/^{206}Pb$*	1s	*$^{207}Pb/^{235}U$*	1s	*$^{206}Pb/^{238}U$*	1s	*$^{207}Pb/^{206}Pb$*	1s	*$^{207}Pb/^{235}U$*	1s	*$^{206}Pb/^{238}U$*	1s	*Th232*	*U238*		
**Ophiolitic gabbro sample 17sat 13-2**																
17sat13-2-01	0.056970	0.001120	0.648220	0.013010	0.082580	0.000800	490	44	507	8	512	5	97.28	371.39	0.26	99%
17sat13-2-02	0.058370	0.001930	0.677960	0.022810	0.084680	0.001030	544	74	526	14	524	6	16.97	84.75	0.20	100%
17sat13-2-03	0.058200	0.001380	0.664820	0.016320	0.082990	0.000920	537	53	518	10	514	5	48.67	213.37	0.23	101%
17sat13-2-04	0.058810	0.002000	0.701700	0.021950	0.087810	0.001240	560	76	540	13	543	7	22.25	90.64	0.25	99%
17sat13-2-05	0.058040	0.001150	0.686020	0.014400	0.085660	0.000840	531	44	530	9	530	5	154.58	355.20	0.44	100%
17sat13-2-06	0.060010	0.002340	0.687290	0.025710	0.083970	0.000960	604	86	531	15	520	6	16.23	87.35	0.19	102%
17sat13-2-07	0.058800	0.001790	0.673760	0.021420	0.083150	0.001070	560	68	523	13	515	6	17.57	93.14	0.19	102%
17sat13-2-08	0.056640	0.000910	0.645200	0.010590	0.082550	0.000690	478	36	506	7	511	4	255.63	537.77	0.48	99%
17sat13-2-09	0.056200	0.001270	0.646110	0.015310	0.083480	0.000860	460	51	506	9	517	5	52.92	250.66	0.21	98%
17sat13-2-10	0.059380	0.001700	0.685580	0.018970	0.084450	0.001020	581	64	530	11	523	6	19.79	111.70	0.18	101%
17sat13-2-11	0.058940	0.001190	0.681610	0.015240	0.083640	0.000880	565	45	528	9	518	5	54.01	248.54	0.22	102%
17sat13-2-12	0.057510	0.001160	0.662210	0.013600	0.083480	0.000830	511	45	516	8	517	5	74.68	248.72	0.30	100%
17sat13-2-13	0.059210	0.001500	0.689120	0.016940	0.084950	0.001020	575	56	532	10	526	6	50.29	194.74	0.26	101%
17sat13-2-14	0.056960	0.001520	0.650680	0.016620	0.083060	0.000760	490	60	509	10	514	5	65.38	214.86	0.30	99%
17sat13-2-15	0.057890	0.001560	0.661850	0.018110	0.082940	0.000940	526	60	516	11	514	6	37.70	217.71	0.17	100%

17sat13-2-16	0.057850	0.001230	0.666670	0.014630	0.082770	0.000880	524	48	519	9	513	5	121.35	328.54	0.37	101%
17sat13-2-17	0.060250	0.002170	0.691850	0.025180	0.083230	0.001060	613	80	534	15	515	6	20.62	94.71	0.22	104%
17sat13-2-18	0.059250	0.001720	0.673500	0.021490	0.081670	0.000850	576	65	523	13	506	5	32.72	113.28	0.29	103%
17sat13-2-19	0.057960	0.001360	0.671460	0.015900	0.083830	0.000730	528	53	522	10	519	4	50.78	239.30	0.21	101%
17sat13-2-20	0.058560	0.001600	0.670150	0.018590	0.082810	0.000880	551	61	521	11	513	5	33.96	171.45	0.20	102%
17sat13-2-21	0.059650	0.001790	0.677730	0.020700	0.082860	0.001070	591	67	525	13	513	6	37.88	115.48	0.33	102%
17sat13-2-22	0.056420	0.001590	0.646990	0.018680	0.082980	0.000930	469	64	507	12	514	6	73.99	225.12	0.33	99%
17sat13-2-23	0.059010	0.001940	0.679220	0.021720	0.083830	0.001040	567	73	526	13	519	6	24.30	92.73	0.26	101%
17sat13-2-24	0.057790	0.002160	0.670150	0.025120	0.084260	0.000980	522	84	521	15	522	6	35.12	87.89	0.40	100%
17sat13-2-25	0.053170	0.001780	0.618820	0.020240	0.084770	0.001050	336	78	489	13	525	6	30.14	131.06	0.23	93%
17sat13-2-26	0.057650	0.002390	0.668280	0.026940	0.084930	0.001250	516	93	520	16	525	7	24.86	75.13	0.33	99%
17sat13-2-27	0.055610	0.001480	0.635500	0.017320	0.082810	0.000930	437	61	500	11	513	6	45.62	234.65	0.19	97%
17sat13-2-28	0.054800	0.001210	0.642380	0.014050	0.084810	0.000760	404	51	504	9	525	5	162.09	443.75	0.37	96%
17sat13-2-29	0.057630	0.001620	0.653240	0.017410	0.082510	0.000860	516	63	510	11	511	5	88.99	221.41	0.40	100%
17sat13-2-30	0.058280	0.003310	0.668620	0.038390	0.083090	0.000970	540	128	520	23	515	6	31.11	157.95	0.20	101%
17sat13-2-31	0.057390	0.001010	0.664700	0.011690	0.083960	0.000740	507	40	517	7	520	4	112.90	375.36	0.30	99%
17sat13-2-32	0.055990	0.001420	0.640060	0.016370	0.082880	0.000860	452	58	502	10	513	5	58.31	194.99	0.30	98%
17sat13-2-33	0.056440	0.001710	0.646620	0.018950	0.083250	0.000910	470	69	506	12	515	5	69.49	217.81	0.32	98%
17sat13-2-34	0.058280	0.002740	0.658170	0.032300	0.081840	0.001320	540	105	513	20	507	8	25.67	92.31	0.28	101%
17sat13-2-35	0.056780	0.001680	0.651580	0.018920	0.083470	0.000990	483	67	509	12	517	6	65.60	169.78	0.39	98%
17sat13-2-36	0.058520	0.001910	0.678080	0.021550	0.084680	0.001090	549	73	526	13	524	6	18.51	110.89	0.17	100%
17sat13-2-37	0.060520	0.001420	0.700510	0.017300	0.083670	0.000810	622	52	539	10	518	5	71.18	199.76	0.36	104%
17sat13-2-38	0.057250	0.001780	0.655600	0.022600	0.082390	0.000960	501	70	512	14	510	6	35.42	135.55	0.26	100%
17sat13-2-39	0.058040	0.001060	0.668520	0.012230	0.083500	0.000800	531	41	520	7	517	5	171.33	359.87	0.48	101%
17sat13-2-40	0.057220	0.001920	0.673690	0.022650	0.085560	0.001040	500	76	523	14	529	6	24.84	122.38	0.20	99%

Table S2. Zircon Hf isotopes of the olivine gabbro within the Munabulake ophiolite

**Ophiolitic gabbro 17sat13-2**																
	t (Ma)	$^{176}Yb/^{177}Hf$	$^{176}Lu/^{177}Hf$	2 s	$^{176}Hf/^{177}Hf$	2 s	I_{Hf}	CHUR	DM	$\varepsilon_{Hf}(t)$	2 s	$f_{Lu/Hf}$	T_{DM1}	2 s	T_{DM2}	2 s
17sat13-2-01	512	0.000618	0.001526	0.000011	0.282830	0.000009	0.282815	0.282463	0.282882	12.5	0.3	-0.95	607	13	662	20
17sat13-2-02	524	0.000114	0.000895	0.000002	0.282821	0.000009	0.282812	0.282455	0.282873	12.6	0.3	-0.97	610	13	662	20
17sat13-2-03	518	0.000132	0.001950	0.000018	0.282863	0.000009	0.282844	0.282459	0.282877	13.6	0.3	-0.94	566	13	593	20
17sat13-2-04	514	0.000569	0.001995	0.000011	0.282814	0.000008	0.282795	0.282461	0.282880	11.8	0.3	-0.94	638	12	706	18
17sat13-2-06	530	0.000128	0.001591	0.000006	0.282840	0.000011	0.282824	0.282451	0.282869	13.2	0.4	-0.95	594	15	630	24
17sat13-2-07	520	0.000182	0.001223	0.000001	0.282831	0.000010	0.282819	0.282458	0.282876	12.8	0.3	-0.96	600	14	648	22
17sat13-2-08	515	0.000170	0.000668	0.000003	0.282848	0.000010	0.282842	0.282461	0.282879	13.5	0.3	-0.98	568	13	600	22
17sat13-2-09	511	0.000541	0.003677	0.000022	0.282813	0.000009	0.282778	0.282463	0.282882	11.1	0.3	-0.89	670	13	747	20
17sat13-2-10	517	0.000295	0.001882	0.000004	0.282806	0.000009	0.282787	0.282459	0.282878	11.6	0.3	-0.94	648	12	722	19
17sat13-2-11	523	0.000232	0.001076	0.000003	0.282835	0.000010	0.282824	0.282456	0.282874	13.1	0.4	-0.97	593	14	634	22
17sat13-2-12	518	0.000416	0.001232	0.000012	0.282811	0.000008	0.282799	0.282459	0.282877	12.1	0.3	-0.96	629	11	694	18
17sat13-2-13	517	0.000192	0.001466	0.000007	0.282838	0.000010	0.282824	0.282459	0.282878	12.9	0.4	-0.96	594	14	639	22
17sat13-2-14	526	0.000229	0.001323	0.000006	0.282800	0.000011	0.282787	0.282454	0.282871	11.8	0.4	-0.96	647	15	718	24
17sat13-2-15	514	0.001400	0.002518	0.000036	0.282846	0.000009	0.282822	0.282461	0.282880	12.8	0.3	-0.92	601	13	647	20
17sat13-2-16	514	0.000185	0.001408	0.000007	0.282838	0.000008	0.282825	0.282461	0.282880	12.9	0.3	-0.96	593	11	639	17
17sat13-2-17	528	0.000204	0.000853	0.000008	0.282820	0.000009	0.282811	0.282452	0.282870	12.7	0.3	-0.97	611	13	661	21
17sat13-2-18	513	0.000157	0.002119	0.000009	0.282808	0.000008	0.282787	0.282462	0.282881	11.5	0.3	-0.94	650	12	725	18
17sat13-2-19	515	0.000198	0.000997	0.000001	0.282803	0.000009	0.282793	0.282461	0.282879	11.8	0.3	-0.97	637	13	710	20
17sat13-2-20	506	0.000182	0.001413	0.000003	0.282792	0.000009	0.282779	0.282466	0.282886	11.1	0.3	-0.96	660	13	749	20
17sat13-2-21	519	0.000107	0.000447	0.000009	0.282835	0.000008	0.282831	0.282458	0.282877	13.2	0.3	-0.99	583	11	623	17

Table S3. Zircon O isotopes and water content of the olivine gabbro within the Munabulake ophiolite

	PI (nA)	*^{16}O/Coeff*	*^{18}O/Coeff*	*$^{16}O^{1}H$/Coeff*	*$^{18}O/^{16}O$*	*SE (%)*	*$^{16}O^{1}H/^{16}O$*	*SE (%)*	*$\delta^{18}O_{correct}$*	*H_2O (ppmw)*
17sat13-2@01	2.6	1.47E+09	2.98E+06	9.57E+04	0.0020247	0.018	6.49E-05	0.08	5.25	327
17sat13-2@02	2.6	1.51E+09	3.06E+06	8.05E+04	0.0020235	0.012	5.32E-05	0.06	4.70	268
17sat13-2@03	2.6	1.50E+09	3.04E+06	2.29E+05	0.0020235	0.015	1.53E-04	0.31	4.70	769
17sat13-2@04	2.6	1.52E+09	3.08E+06	7.27E+04	0.0020254	0.015	4.78E-05	0.08	5.69	241
17sat13-2@05	2.6	1.54E+09	3.11E+06	1.68E+05	0.0020250	0.011	1.09E-04	0.07	5.53	549
17sat13-2@06	2.6	1.55E+09	3.14E+06	7.95E+04	0.0020240	0.009	5.13E-05	0.13	5.13	258
17sat13-2@07	2.5	1.50E+09	3.04E+06	9.86E+04	0.0020255	0.015	6.56E-05	0.38	5.90	331
17sat13-2@08	2.6	1.55E+09	3.14E+06	2.98E+05	0.0020230	0.013	1.92E-04	0.09	4.69	968
17sat13-2@09	2.5	1.57E+09	3.18E+06	2.35E+05	0.0020232	0.018	1.49E-04	0.54	4.81	751
17sat13-2@10	2.6	1.56E+09	3.16E+06	4.83E+04	0.0020221	0.015	3.09E-05	0.16	4.31	155
17sat13-2@11	2.5	1.55E+09	3.13E+06	1.67E+05	0.0020233	0.022	1.08E-04	0.21	5.27	545
17sat13-2@12	2.5	1.55E+09	3.13E+06	3.66E+05	0.0020246	0.014	2.37E-04	0.09	4.94	1192
17sat13-2@13	2.5	1.51E+09	3.05E+06	6.83E+04	0.0020237	0.012	4.53E-05	0.08	5.61	228
17sat13-2@14	2.5	1.50E+09	3.03E+06	4.81E+04	0.0020248	0.014	3.21E-05	0.54	4.73	162
17sat13-2@15	2.6	1.54E+09	3.11E+06	2.89E+05	0.0020248	0.016	1.88E-04	0.04	4.73	946
17sat13-2@16	2.5	1.52E+09	3.08E+06	5.44E+04	0.0020244	0.017	3.57E-05	0.12	4.60	180
17sat13-2@17	2.6	1.50E+09	3.03E+06	3.98E+05	0.0020257	0.012	2.66E-04	0.40	5.31	1339
17sat13-2@18	2.6	1.52E+09	3.09E+06	1.46E+05	0.0020255	0.017	9.59E-05	0.15	5.21	483
17sat13-2@19	2.5	1.53E+09	3.10E+06	2.07E+05	0.0020241	0.014	1.35E-04	0.17	4.58	680

17sat13-2@20	2.8	1.50E+09	3.04E+06	1.86E+05	0.0020243	0.014	1.24E-04	0.07	4.69	624
~~17sat13-2@21~~	~~2.6~~	~~1.53E+09~~	~~3.10E+06~~	~~3.31E+04~~	~~0.0020201~~	~~0.018~~	~~2.16E-05~~	~~0.17~~	~~2.69~~	~~109~~
17sat13-2@22	2.6	1.50E+09	3.05E+06	1.47E+05	0.0020254	0.018	9.80E-05	0.09	5.36	494
17sat13-2@23	2.6	1.50E+09	3.04E+06	1.88E+05	0.0020254	0.012	1.25E-04	0.13	5.38	631
17sat13-2@24	2.6	1.54E+09	3.12E+06	1.81E+05	0.0020242	0.016	1.18E-04	0.15	4.82	592
17sat13-2@25	2.7	1.52E+09	3.08E+06	1.54E+05	0.0020240	0.016	1.01E-04	0.35	4.76	510
17sat13-2@26	2.6	1.49E+09	3.02E+06	1.59E+05	0.0020254	0.016	1.07E-04	0.18	5.53	536
17sat13-2@27	2.6	1.50E+09	3.05E+06	3.97E+04	0.0020254	0.011	2.64E-05	0.28	5.58	133
17sat13-2@28	2.6	1.50E+09	3.03E+06	1.53E+05	0.0020247	0.013	1.03E-04	0.10	5.26	516
17sat13-2@29	2.7	1.53E+09	3.10E+06	1.57E+05	0.0020253	0.015	1.02E-04	0.22	5.58	516
17sat13-2@30	2.5	1.56E+09	3.15E+06	7.86E+04	0.0020247	0.016	5.05E-05	0.23	5.34	254
17sat13-2@31	2.5	1.51E+09	3.06E+06	7.42E+04	0.0020255	0.010	4.92E-05	0.14	5.67	248
17sat13-2@32	2.8	1.51E+09	3.07E+06	8.79E+04	0.0020242	0.015	5.80E-05	0.37	5.03	292
17sat13-2@33	2.5	1.51E+09	3.06E+06	1.34E+05	0.0020241	0.014	8.88E-05	0.13	4.99	447
17sat13-2@34	2.5	1.50E+09	3.04E+06	1.81E+05	0.0020251	0.011	1.20E-04	0.19	5.48	605
17sat13-2@35	2.6	1.47E+09	2.97E+06	7.82E+04	0.0020252	0.017	5.34E-05	0.26	5.52	269
17sat13-2@36	2.6	1.50E+09	3.05E+06	1.83E+05	0.0020248	0.015	1.22E-04	0.11	5.34	614
17sat13-2@37	2.6	1.48E+09	3.00E+06	5.96E+04	0.0020253	0.012	4.02E-05	0.14	5.59	202
17sat13-2@38	2.7	1.56E+09	3.15E+06	8.93E+04	0.0020236	0.010	5.73E-05	0.16	4.72	289
17sat13-2@39	2.6	1.51E+09	3.05E+06	4.89E+04	0.0020196	0.021	3.24E-05	0.26	3.25	163
17sat13-2@40	2.6	1.55E+09	3.13E+06	9.98E+04	0.0020205	0.012	6.45E-05	0.15	3.70	325

Table S4. Whole rock geochemical data for rock suites of the Munabulake ophiolite.

Sample name	17SAT13-2	17SAT31	17SAT8	17SAT10	18SAT36-3	17SAT11-2(2)	18SAT32-1	17SAT9	17SAT3-1	17SAT11-2(1)	17SAT3-2	18SAT33-2
SiO2	45.96	48.58	49.90	49.22	50.42	50.52	52.51	52.58	54.22	54.05	55.00	55.42
TiO2	0.73	0.10	0.31	0.83	0.42	0.43	0.42	0.68	1.12	0.78	1.76	0.45
Al2O3	14.80	22.15	18.43	16.24	15.87	17.59	17.20	15.99	15.19	14.52	13.83	17.21
Fe2O3	12.36	6.69	9.86	12.19	11.68	10.62	6.70	10.26	13.13	13.23	13.03	10.38
MnO	0.29	0.09	0.23	0.20	0.19	0.18	0.21	0.18	0.26	0.22	0.22	0.22
MgO	7.40	9.95	6.63	7.69	7.91	7.07	5.12	6.80	4.93	5.27	3.75	4.97
CaO	17.28	9.17	9.82	9.93	11.47	9.53	14.93	8.35	6.19	7.88	7.36	6.44
Na2O	0.71	3.12	3.41	3.32	1.87	3.61	1.32	4.34	4.66	3.69	4.70	4.22
K2O	0.43	0.14	1.41	0.31	0.12	0.40	1.53	0.75	0.19	0.27	0.17	0.63
P2O5	0.06	0.02	0.02	0.09	0.04	0.04	0.06	0.05	0.10	0.08	0.20	0.06
Mg#	54.49	74.84	57.35	55.79	57.53	57.11	60.45	57.00	42.89	44.34	36.53	48.92
Sum	100.00	100.00	100.00	100.00	100.00	100.00	100.00	100.00	100.00	100.00	100.00	100.00
**Li**	12.70	2.03	22.7	14.76	10.1	8.99	5.25	13.78	13.42	3.94	7.53	14.5
**Be**	0.45	0.22	0.31	0.35	0.32	0.43	0.48	0.36	0.46	0.40	0.70	0.35
**Sc**	46	2.52	35	39	37.4	40	33.4	35	32	40	27.4	34.5
**Ti**	4138	611	1859	4890	2513	2492	2499	3943	6479	4580	10359	2577
**V**	325	11.87	187	303	241	233	168	260	355	327	280	231
**Cr**	1469	2.25	192	292	227	49	277	97	11.84	10.83	4.28	17.2
**Mn**	2190	671	1723	1522	1452	1362	1689	1298	1826	1605	1560	1682
**Co**	48	58	43	44	46.4	43	28.4	38	39	41	29.2	33.3
**Ni**	237	77	53	88	67.2	50	69.3	46	21.4	16.68	6.42	19.8
**Cu**	3.87	24.1	2.63	9.21	1.43	78	83.3	54	59	2.62	36	1.62

**Zn**	82	36	125	97	40.9	89	66.3	73	113	66	105	159
**Ga**	12.34	11.55	11.55	14.57	13.4	12.10	14.8	13.01	15.04	13.22	16.30	14.3
**As**	5.76	2.66	1.32	1.36	0.50	0.99	0.28	1.25	0.86	1.96	1.92	0.36
**Se**	0.21	0.08	0.28	0.72	0.21	0.36	0.34	0.53	0.85	0.67	0.91	0.23
**Rb**	27.9	0.55	75	10.44	3.16	8.39	56.0	27.4	3.76	1.86	2.03	16.6
**Sr**	164	819	348	123	123	156	333	438	128	55	89	89.1
**Rb/Sr**	0.17	0.00	0.22	0.09	0.03	0.05	0.17	0.06	0.03	0.03	0.02	0.19
**Y**	12.57	0.68	7.62	19.53	12.4	12.44	16.2	18.54	25.6	18.96	40	12.8
**Zr**	41	2.46	12.38	37	19.2	22.8	19.3	33	59	36	105	28.9
**Nb**	1.89	0.11	0.31	2.22	0.88	1.29	0.86	1.19	2.29	1.77	4.99	1.14
**Mo**	0.25	1.40	0.31	0.21	0.02	0.90	0.61	0.28	0.31	1.26	0.54	0.08
**Cs**	12.76	0.02	2.54	0.79	0.66	0.40	4.50	1.82	2.64	0.63	0.69	1.23
**Ba**	16.89	45	198	61	68.3	216	475	391	64	18.37	67	83.0
**La**	6.39	1.31	1.57	3.63	1.67	1.46	2.71	3.34	4.03	3.61	8.61	1.59
**Ce**	12.41	2.69	3.07	8.47	4.04	3.38	5.65	7.48	10.08	8.52	20.8	4.07
**Pr**	1.56	0.34	0.51	1.22	0.58	0.52	0.74	1.07	1.57	1.26	3.14	0.61
**Nd**	6.15	1.29	2.37	5.91	2.87	2.64	3.66	5.09	7.87	5.89	15.16	3.20
**Sm**	1.56	0.23	0.78	1.95	0.96	1.00	1.25	1.67	2.62	1.84	4.47	1.12
**Eu**	0.56	0.45	0.43	0.76	0.46	0.36	0.59	0.63	0.89	0.68	1.60	0.40
**Gd**	1.83	0.19	1.09	2.70	1.45	1.45	1.76	2.32	3.46	2.46	5.65	1.62
**Tb**	0.32	0.02	0.19	0.48	0.27	0.27	0.34	0.44	0.63	0.45	1.01	0.29
**Dy**	2.09	0.13	1.32	3.25	1.98	1.99	2.48	3.07	4.37	3.15	6.73	2.06
**Ho**	0.48	0.02	0.28	0.71	0.44	0.46	0.55	0.68	0.95	0.70	1.44	0.45
**Er**	1.39	0.06	0.82	2.09	1.34	1.38	1.70	1.99	2.80	2.06	4.30	1.32
**Tm**	0.20	0.01	0.13	0.33	0.21	0.22	0.27	0.31	0.42	0.32	0.64	0.21
**Yb**	1.34	0.07	0.86	2.21	1.37	1.48	1.75	2.04	2.78	2.08	4.17	1.33

**Lu**	0.21	0.01	0.13	0.35	0.21	0.24	0.27	0.31	0.44	0.33	0.64	0.21
**Hf**	1.09	0.05	0.42	1.25	0.64	0.77	0.64	1.08	1.84	1.16	3.07	0.88
**Ta**	0.12	0.02	0.02	0.13	0.06	0.11	0.04	0.07	0.15	0.10	0.26	0.04
**Pb**	5.98	0.49	4.80	5.52	3.13	11.52	14.9	32	5.19	2.50	6.15	5.60
**Th**	1.53	0.05	0.10	1.10	0.43	0.64	0.55	0.72	0.90	1.00	1.37	0.26
**U**	0.46	0.02	0.16	0.36	0.23	0.21	0.96	0.35	0.46	1.01	0.62	0.23

Sample name	17SAT2-1	17SAT11-4	18SAT36-2	17SAT5	17SAT6	17SAT12	18SAT41-4	18SAT41-5	17SAT13-1	17SAT14	17SAT22-1
SiO2	56.24	55.20	55.37	55.47	56.51	58.54	43.52	48.05	44.51	44.40	43.81
TiO2	0.63	0.65	1.18	0.92	1.24	0.36	0.01	0.01	0.00	0.00	0.00
Al2O3	15.10	15.57	14.92	15.00	15.06	14.91	0.43	1.14	0.58	0.61	0.29
Fe2O3	11.42	12.04	13.39	10.22	12.27	7.21	9.38	8.22	9.55	9.12	8.98
MnO	0.18	0.19	0.17	0.20	0.19	0.15	0.12	0.12	0.11	0.14	0.14
MgO	4.75	4.91	3.54	4.79	3.91	4.53	45.76	42.32	45.07	45.51	46.27
CaO	8.25	7.62	8.53	9.30	5.73	12.42	0.70	0.07	0.17	0.21	0.50
Na2O	3.04	3.57	2.70	3.59	4.83	0.99	0.07	0.04	0.00	0.00	0.00
K2O	0.32	0.18	0.08	0.40	0.15	0.86	0.00	0.01	0.00	0.01	0.00
P2O5	0.07	0.06	0.12	0.11	0.10	0.03	0.02	0.01	0.01	0.01	0.01
Mg#	45.41	44.92	34.59	48.38	38.92	55.69	90.70	91.15	90.42	90.89	91.15
Sum	100.00	100.00	100.00	100.00	100.00	100.00	100.00	100.00	100.00	100.00	100.00
**Li**	5.17	6.11	13.0	4.06	16.19	1.68	11.3	14.0	22.8	0.80	1.74
**Be**	0.41	0.56	0.46	0.26	0.56	0.25	0.47	1.52	0.16	0.01	-
**Sc**	36	37	32.1	29.9	35	28.8	7.46	7.42	19.73	7.05	6.74
**Ti**	3518	3825	6847	5263	7229	2121	34.4	135	1665	17.30	12.04
**V**	296	314	347	206	366	240	24.6	36.2	144	27.6	26.4

**Cr**	20.3	16.36	4.35	25.7	0.79	64	2589	2581	2476	2081	2441
**Mn**	1313	1421	1218	1462	1433	1136	794	786	786	795	844
**Co**	35	39	36.1	33	35	21.3	103	103	38	104	110
**Ni**	22.8	19.93	9.26	21.0	3.34	35	2203	2216	439	2278	2408
**Cu**	0.19	99	55.2	0.91	50	15.71	3.19	8.45	2.36	1.79	3.40
**Zn**	34	78	53.6	111	96	38	91.3	65.8	48	36	39
**Ga**	13.13	13.48	15.6	12.85	15.25	15.88	1.43	1.86	5.31	0.39	0.32
**As**	0.82	0.73	0.67	0.91	1.64	1.01	1.61	13.4	2.39	0.33	-
**Se**	0.48	0.40	0.59	0.53	0.93	0.44	0.03	0.09	0.18	0.10	0.15
**Rb**	1.51	1.31	2.87	14.81	5.15	36	3.77	56.4	0.59	0.25	0.32
**Sr**	70	140	169	104	100	200	2.58	13.2	126	5.23	1.38
**Rb/Sr**	0.02	0.01	0.02	0.14	0.05	0.18	1.46	4.27	0.00	0.05	0.23
**Y**	16.50	16.49	27.1	24.1	32	12.35	0.40	2.03	4.60	0.08	0.08
**Zr**	29.7	32	63.4	62	84	18.49	0.59	5.06	18.46	0.20	0.23
**Nb**	1.44	1.56	2.16	0.67	0.77	0.74	0.47	0.89	2.88	0.06	0.04
**Mo**	0.58	0.61	0.12	0.46	0.45	0.48	0.13	0.14	0.14	0.23	0.14
**Cs**	1.00	0.17	0.59	0.72	0.56	4.63	8.79	49.1	0.82	0.14	0.07
**Ba**	18.26	24.2	51.5	78	53	300	2.57	2.89	35	10.23	4.59
**La**	2.99	3.18	3.99	2.46	3.22	2.09	0.34	1.93	4.41	0.06	0.10
**Ce**	7.04	6.86	10.1	7.47	9.70	4.34	0.63	4.43	8.76	0.12	0.19
**Pr**	1.00	1.02	1.48	1.29	1.72	0.64	0.06	0.57	1.09	0.02	0.02
**Nd**	4.73	4.78	7.49	6.91	9.30	3.02	0.20	2.33	4.38	0.06	0.09
**Sm**	1.47	1.55	2.60	2.46	3.30	0.99	0.04	0.46	0.92	0.01	0.02
**Eu**	0.55	0.55	0.90	0.93	1.17	0.47	0.00	0.18	0.26	0.00	0.00
**Gd**	1.97	2.06	3.51	3.32	4.36	1.47	0.04	0.40	0.96	0.01	0.01
**Tb**	0.37	0.38	0.65	0.59	0.78	0.28	0.01	0.06	0.14	0.00	0.00

**Dy**	2.66	2.69	4.58	4.12	5.35	2.03	0.06	0.37	0.83	0.01	0.01
**Ho**	0.59	0.60	1.00	0.89	1.16	0.46	0.01	0.07	0.17	0.00	0.00
**Er**	1.79	1.81	2.92	2.66	3.42	1.35	0.05	0.21	0.45	0.01	0.01
**Tm**	0.27	0.28	0.45	0.39	0.52	0.21	0.01	0.03	0.07	0.00	0.00
**Yb**	1.85	1.86	2.92	2.56	3.28	1.38	0.08	0.20	0.40	0.01	0.02
**Lu**	0.29	0.30	0.47	0.39	0.49	0.21	0.02	0.03	0.06	0.00	0.00
**Hf**	0.97	1.03	1.87	1.83	2.57	0.64	0.01	0.11	0.47	0.01	0.01
**Ta**	0.09	0.10	0.14	0.07	0.05	0.05	0.09	0.04	0.18	0.01	0.01
**Pb**	1.59	5.77	1.79	2.79	2.35	8.25	0.22	0.74	0.98	0.30	0.29
**Th**	0.83	1.01	0.92	0.31	0.34	0.50	0.21	0.28	1.06	0.03	0.04
**U**	0.72	0.45	0.45	1.36	0.28	0.21	0.76	0.59	0.79	0.02	0.05

Table S5. Mineral compositions of spinels and olivines within peridotite of the Munabulake ophiolite.

**Spinel**	MgO	SiO2	Na2O	Al2O3	NiO	FeO	Cr2O3	CaO	K2O	P2O5	TiO2	Total	Mg#	Cr#
18JL41-4Sp-1	5.44	0.09	0.04	7.94	0.00	27.74	55.78	0.00	0.01	0.00	0.20	97.23	26.08	82.51
18JL41-4Sp-2	6.44	0.03	0.01	11.36	0.08	25.06	55.34	0.00	0.00	0.02	0.11	98.43	31.61	76.57
18JL41-4Ol-Sp-1	2.61	0.02	0.01	3.01	0.00	33.16	56.54	0.00	0.00	0.02	0.17	95.53	12.42	92.65
18JL41-4Ol-Sp-1	4.90	0.02	0.07	13.32	0.00	28.80	49.18	0.00	0.02	0.00	0.19	96.50	23.42	71.25
18JL41-4Ol-Sp-1	4.55	0.01	0.03	9.16	0.00	28.23	55.27	0.00	0.00	0.01	0.12	97.38	22.48	80.19
18JL41-4Ol-Sp-2	5.13	0.00	0.08	13.30	0.04	27.28	50.50	0.00	0.01	0.01	0.14	96.48	25.28	71.82
18JL41-4Ol-Sp-1	5.21	0.35	0.02	10.50	0.16	29.04	53.26	0.00	0.03	0.03	0.04	98.63	24.41	77.30
18JL41-4Ol-Sp-2	4.58	0.00	0.07	7.47	0.09	27.90	57.74	0.00	0.02	0.00	0.13	97.99	22.81	83.83
18JL41-4Ol-Sp-3	5.36	0.00	0.07	12.98	0.01	28.66	50.51	0.00	0.00	0.00	0.07	97.65	25.17	72.32
18JL41-4Ol-Sp-4	4.83	0.00	0.10	11.18	0.16	28.57	51.98	0.00	0.00	0.02	0.19	97.03	23.35	75.73
18JL41-4Ol-Sp-5	4.86	0.05	0.02	11.52	0.05	28.13	52.01	0.00	0.00	0.00	0.14	96.77	23.72	75.19
22-1Ol-Sp-1	6.99	0.00	0.02	17.90	0.00	24.58	48.93	0.00	0.00	0.01	0.00	98.42	33.87	64.71
22-1Ol-Sp-1	7.16	0.07	0.08	19.57	0.18	24.16	48.48	0.02	0.00	0.00	0.01	99.72	34.77	62.44
22-1Ol-Sp-1	7.30	0.03	0.03	20.43	0.00	23.91	45.93	0.03	0.00	0.00	0.00	97.64	35.45	60.14
22-1Ol-Sp-2	6.96	0.02	0.03	17.77	0.04	24.19	49.12	0.00	0.00	0.01	0.00	98.14	34.13	64.98
22-1Ol-Sp-1	6.60	0.05	0.03	17.07	0.03	24.76	48.43	0.00	0.00	0.00	0.03	96.98	32.42	65.57
22-1Ol-Sp-3	7.06	0.05	0.05	19.53	0.08	23.73	45.04	0.00	0.02	0.00	0.03	95.58	34.88	60.75
22-1Ol-Sp-1	7.38	0.01	0.02	20.28	0.02	23.71	46.56	0.00	0.01	0.00	0.05	98.05	35.92	60.64
18JL41-5Ol-Sp-1	12.44	0.04	0.14	38.73	0.49	19.34	26.92	0.13	0.07	0.04	0.03	98.38	53.67	31.81
18JL41-5Ol-Sp-2	13.35	0.06	0.03	42.11	0.08	17.98	26.98	0.01	0.00	0.04	0.03	100.66	57.21	30.07
18JL41-5Ol-Sp-1	11.95	0.04	0.01	37.26	0.10	18.77	27.44	0.02	0.01	0.00	0.12	95.71	53.40	33.08
18JL41-5Ol-Sp-2	13.99	0.00	0.00	42.98	0.13	16.32	22.22	0.00	0.00	0.03	0.02	95.69	60.68	25.76
18JL41-5Ol-Sp-1	8.09	0.03	0.07	25.92	0.11	24.54	36.42	0.00	0.01	0.02	0.06	95.26	37.25	48.53
18JL41-5Ol-Sp-1	9.11	0.02	0.02	30.77	0.10	23.15	34.42	0.00	0.00	0.00	0.04	97.63	41.46	42.88

18JL41-5Ol-Sp-3	9.37	0.12	0.05	29.45	0.01	23.80	35.17	0.01	0.02	0.00	0.12	98.12	41.47	44.48
18JL41-5Ol-Sp-4	9.56	0.07	0.03	30.32	0.04	22.70	35.32	0.00	0.00	0.01	0.08	98.12	43.11	43.88
18JL41-5Ol-Sp-5	9.18	0.06	0.07	28.43	0.06	22.23	34.47	0.10	0.02	0.01	0.04	94.67	42.65	44.86
18JL41-5Ol-Sp-1	9.22	0.10	0.05	27.71	0.05	23.71	36.09	0.00	0.01	0.00	0.09	97.02	41.17	46.64
18JL41-5Ol-Sp-1	6.59	0.00	0.00	16.03	0.00	26.61	50.08	0.01	0.01	0.00	0.14	99.47	30.82	67.70
18JL41-5Ol-Sp-2	7.31	0.03	0.02	17.51	0.00	25.15	49.45	0.00	0.01	0.00	0.04	99.53	34.34	65.46
18JL41-5Ol-Sp-3	8.43	0.02	0.04	22.29	0.02	24.57	41.45	0.00	0.03	0.00	0.12	96.96	38.19	55.51
18JL41-5Ol-Sp-4	6.80	0.04	0.02	16.29	0.00	25.83	49.30	0.00	0.00	0.00	0.06	98.34	32.16	67.01
18JL41-5Ol-Sp-5	8.53	0.00	0.05	22.11	0.01	25.49	41.35	0.00	0.01	0.00	0.00	97.54	37.60	55.65
18JL41-5Ol-Sp-1	3.98	0.04	0.08	10.27	0.05	30.51	50.69	0.00	0.05	0.02	0.09	95.79	19.00	76.82
18JL41-5Ol-Sp-1	14.26	0.03	0.06	44.41	0.01	15.45	22.57	0.00	0.00	0.00	0.07	96.84	62.42	25.43
18JL41-5Ol-Sp-1	10.84	0.02	0.00	35.05	0.00	20.92	31.55	0.00	0.01	0.02	0.00	98.42	48.27	37.66
18JL41-5Ol-Sp-3	6.52	0.07	0.11	17.64	0.01	24.93	48.34	0.00	0.01	0.00	0.00	97.61	32.00	64.78

**Olivine**	MgO	SiO2	Na2O	Al2O3	NiO	FeO	Cr2O3	CaO	K2O	P2O5	TiO2	Total	Fo
18JL41-4Ol-1	48.97	41.10	0.02	0.00	0.29	8.71	0.02	0.00	0.00	0.00	0.00	99.11	91.0
18JL41-4Ol-2	48.41	40.95	0.00	0.00	0.43	8.60	0.02	0.00	0.00	0.00	0.01	98.41	91.0
18JL41-4Ol-Sp-2	49.27	41.27	0.01	0.02	0.33	8.85	0.04	0.00	0.00	0.03	0.04	99.85	90.9
18JL41-4Ol-Sp-2	49.99	41.61	0.01	0.02	0.39	8.86	0.05	0.01	0.00	0.00	0.00	100.94	91.0
18JL41-4Ol-Sp-3	49.39	41.45	0.04	0.00	0.26	8.78	0.11	0.00	0.01	0.01	0.00	100.04	91.0
18JL41-4Ol-Sp-4	48.99	40.82	0.01	0.00	0.44	8.56	0.04	0.00	0.00	0.02	0.07	98.95	91.1
18JL41-4Ol-Sp-6	48.87	41.44	0.00	0.04	0.29	8.84	0.00	0.00	0.01	0.00	0.05	99.55	90.9
18JL41-4Ol-Sp-7	48.70	41.08	0.01	0.00	0.26	8.71	0.02	0.00	0.01	0.02	0.00	98.80	91.0
18JL41-4Ol-Sp-8	48.98	41.59	0.00	0.01	0.52	8.76	0.02	0.00	0.01	0.01	0.01	99.91	91.0
18JL41-4Ol-Sp-9	49.31	41.69	0.00	0.00	0.34	8.06	0.00	0.01	0.02	0.01	0.00	99.44	91.7
22-1Ol-Sp-2	49.12	40.73	0.03	0.02	0.28	7.78	0.00	0.01	0.00	0.01	0.00	97.99	91.9
22-1Ol-Sp-2	50.48	42.25	0.05	0.01	0.35	8.49	0.03	0.00	0.02	0.01	0.05	101.75	91.5

22-1Ol-Sp-2	49.28	41.59	0.00	0.04	0.38	8.34	0.00	0.00	0.01	0.03	0.00	99.67	91.4
22-1Ol-Sp-2	48.84	41.00	0.02	0.00	0.46	8.81	0.07	0.01	0.01	0.00	0.03	99.25	90.9
22-1Ol-Sp-2	49.45	41.18	0.00	0.00	0.43	8.35	0.00	0.01	0.00	0.00	0.00	99.41	91.4
22-1Ol-Sp-1	49.85	41.97	0.04	0.00	0.46	8.60	0.02	0.01	0.00	0.00	0.00	100.97	91.3
22-1Ol-Sp-2	48.91	40.60	0.07	0.02	0.34	8.68	0.00	0.03	0.01	0.00	0.00	98.67	91.0
22-1Ol-Sp-4	49.51	41.14	0.02	0.00	0.50	8.44	0.00	0.00	0.00	0.00	0.00	99.63	91.3
22-1Ol-Sp-2	48.77	40.93	0.01	0.00	0.46	8.48	0.02	0.01	0.00	0.00	0.01	98.69	91.2
18JL41-5Ol-Sp-3	47.46	40.10	0.01	0.00	0.39	9.26	0.02	0.01	0.01	0.00	0.00	97.25	90.2
18JL41-5Ol-Sp-4	48.75	41.63	0.00	0.00	0.27	9.88	0.00	0.00	0.02	0.00	0.00	100.55	89.9
18JL41-5Ol-Sp-3	48.93	40.96	0.02	0.02	0.34	9.47	0.06	0.01	0.01	0.00	0.00	99.81	90.3
18JL41-5Ol-Sp-2	50.28	42.82	0.01	0.00	0.27	8.83	0.06	0.02	0.00	0.01	0.10	102.41	91.1
18JL41-5Ol-Sp-2	49.03	41.10	0.01	0.00	0.43	8.92	0.05	0.01	0.00	0.01	0.00	99.57	90.8
18JL41-5Ol-Sp-7	49.54	41.44	0.00	0.00	0.33	9.33	0.05	0.00	0.01	0.01	0.03	100.73	90.5
18JL41-5Ol-Sp-8	47.64	39.98	0.03	0.00	0.35	9.19	0.00	0.00	0.01	0.00	0.00	97.19	90.3
18JL41-5Ol-Sp-2	49.11	40.68	0.01	0.00	0.38	9.42	0.01	0.00	0.00	0.01	0.00	99.62	90.4
18JL41-5Ol-Sp-2	50.02	41.83	0.02	0.00	0.37	8.85	0.06	0.00	0.01	0.00	0.00	101.15	91.0
18JL41-5Ol-Sp-6	49.30	41.69	0.04	0.00	0.35	9.19	0.03	0.00	0.02	0.01	0.05	100.68	90.6
18JL41-5Ol-Sp-7	48.71	41.12	0.02	0.00	0.32	8.85	0.04	0.01	0.01	0.02	0.01	99.11	90.8
18JL41-5Ol-Sp-8	50.00	41.60	0.02	0.01	0.38	8.93	0.15	0.00	0.00	0.00	0.03	101.13	91.0
18JL41-5Ol-Sp-9	49.43	41.01	0.04	0.00	0.27	8.53	0.51	0.00	0.00	0.00	0.02	99.79	91.3
18JL41-5Ol-Sp-10	49.11	41.43	0.02	0.06	0.25	8.56	0.11	0.00	0.02	0.00	0.02	99.59	91.2
18JL41-5Ol-Sp-2	49.25	40.82	0.00	0.00	0.41	9.52	0.02	0.00	0.01	0.01	0.00	100.05	90.3
18JL41-5Ol-Sp-2	50.32	42.11	0.02	0.00	0.33	9.59	0.00	0.00	0.01	0.02	0.01	102.41	90.4
18JL41-5Ol-Sp-2	49.29	41.27	0.00	0.00	0.34	8.87	0.07	0.00	0.00	0.02	0.06	99.92	90.9
